# Host‐Microbiome Associations of Native and Invasive Small Mammals Across a Tropical Urban–Rural Ecotone

**DOI:** 10.1111/mec.17782

**Published:** 2025-04-28

**Authors:** Alessandra Giacomini, Maklarin B. Lakim, Fred Y. Y. Tuh, Matthew Hitchings, Sofia Consuegra, Tamsyn Uren Webster, Konstans Wells

**Affiliations:** ^1^ Department of Biosciences Swansea University Swansea UK; ^2^ Sabah Parks Kota Kinabalu Sabah Malaysia; ^3^ Institute of Life Science Swansea University Swansea UK; ^4^ Laboratorio de Biotecnología Acuática Instituto de Investigaciones Marinas (IIM‐CSIC) Vigo Spain

**Keywords:** gut microbiota, invasive rats, microbial community ecology, native‐invasive species interactions, phylosymbiosis, urban adaptation

## Abstract

Global change and urbanisation profoundly alter wildlife habitats, driving native animals into novel habitats while increasing the co‐occurrence between native and invasive species. Host‐microbiome associations are shaped by host traits and environmental features, but little is known about their plasticity in co‐occurring native and invasive species across urban–rural gradients. Here, we explored gut microbiomes of four sympatric small mammal species along an urban–rural ecotone in Borneo, one of the planet's oldest rainforest regions experiencing recent urban expansion. Host species identity was the strongest determinant of microbiome composition, while land use and spatial proximity shaped microbiome similarity within and among the three rat species. The urban‐dwelling rat *Rattus rattus* had a microbiome composition more similar to that of the native, urban‐adapted rat *Sundamys muelleri* ( *R. rattus* ' strongest environmental niche overlap), than to the closely related urban‐dwelling *R. norvegicus* . The urban‐dwelling shrew *Suncus murinus* presented the most distinct microbiome. The microbiome of *R. norvegicus* was the most sensitive to land use intensity, exhibiting significant alterations in composition and bacterial abundance across the ecotone. Our findings suggest that environmental niche overlap among native and invasive species promotes similar gut microbiomes. Even for omnivorous urban‐dwellers with a worldwide distribution like *R. norvegicus* , gut microbiomes may change across fine‐scale environmental gradients. Future research needs to confirm whether land use intensity can be a strong selective force on mammalian gut microbiomes, influencing the way in which native and invasive species are able to exploit novel environments.

## 1 Introduction

Human‐induced habitat alteration and encroachment present ever increasing threats to wild animals, affecting both physical and biotic environments and compromising habitat suitability and availability (Alberti 2015; McKinney 2002; Otto 2018). To avoid extinction, native species are often forced to constrain their distribution into the remaining habitat or adapt to new anthropized environments (here, defined as urban adapters) (McKinney 2002; Otto 2018). Urban sprawl also facilitates the introduction and establishment of urban‐dwelling species (Bradley and Altizer 2007; Hassell et al. 2017). These include commensal, non‐native and invasive species capable of thriving in heavily modified landscapes and highly adaptable to changing environmental conditions (Borden and Flory 2021; Hulme‐Beaman et al. 2016). Consequently, habitat encroachment and urban sprawl likely result in the co‐occurrence of urban‐adapted native species and urban‐dwelling non‐native species, promoting changes in community composition and creating new contact opportunities within and between species. Urban–rural ecotones offer fascinating ‘natural laboratories’ to gain insights into the co‐occurrence of species with different life histories and levels of adaptation to anthropized environments, including their associations with symbiotic species such as parasites and microbial communities, which significantly impact their health and survival. Both environmental conditions as well as the interactions among coexisting species may exert selective pressures or offer novel opportunities for the formation of associations of host species with parasites and microbes (Clark et al. 2018; Raulo et al. 2024; Teng et al. 2022). Therefore, exploring the plasticity in microbiome associations of native and invasive species that co‐occur across habitat ecotones offers a multi‐host perspective of how selective forces (i.e., environmental constraint and host traits) and ecological opportunities arising from new contact opportunities among host species may shape host‐microbiome associations.

Animal gut microbiomes play a critical role in maintaining the health and homeostasis of their hosts (Mcfall‐Ngai et al. 2013). While significant disruption in microbiome structure and diversity, known as dysbiosis, can have negative effects on animal health and fitness, the compositional and functional plasticity of the microbiome can also contribute to the host's adaptability to environmental changes (Alberdi et al. 2016). Understanding the factors that can alter the host‐associated microbiome, and how they do so, can therefore provide valuable insights into the impact of different environmental conditions on animals and their capacity for adaptation. Generally, the host's evolutionary history and diet are considered to be the main drivers of gut microbiome composition and diversity, although the debate around their respective roles and degree of influence is still open (Mallott and Amato 2021; Youngblut et al. 2019). Host phylogeny has been widely reported as a major modulator of gut microbiome composition in mammals (Brooks et al. 2016; de Jonge et al. 2022; Mazel et al. 2023). In addition, major diet shifts during the evolution and radiation of mammals have likely shaped gut microbiomes, and it has been suggested that host phylogeny relates to the acquisition of more recently diverged microbial lineages, while host diet relates to the association with large groups of more ancient microbial lineages (Groussin et al. 2017). Strong diet specialisation could enhance host‐microbiome coadaptation (Ley et al. 2008), while opportunistic feeding on certain diet types may increase or reduce microbiome diversity and favour certain bacterial taxa (Davidson et al. 2020; Li et al. 2023; Youngblut et al. 2019). More broadly, a host species realised ecological niche, including preferred habitat, diet, as well as intra‐ and interspecific interactions among host individuals, can be expected to shape the variation in microbial associations found in different host species in any given environment. For example, social behaviours increase the microbial dispersion between individuals, while sharing a common environment can indirectly facilitate the transfer of gut microorganisms among both conspecifics and heterospecifics (Raulo et al. 2024; Sarkar et al. 2020; Stothart et al. 2021; Teng et al. 2022).

Anthropogenic pressures that dramatically alter local habitats and host species community assembly can be expected to have fundamental effects on the diversity and composition of the gut microbiome. Studies focusing on single host species, for example, have demonstrated that human‐induced land‐use changes, such as habitat loss, fragmentation, and urbanisation, are altering gut microbiomes across a diversity of vertebrate species (Berlow et al. 2021; Fackelmann et al. 2021; Maraci et al. 2022; Stothart et al. 2019; Teyssier et al. 2018). Recent research exploring microbiome variation of multiple small mammal host species has further shown that environmental conditions may impose different selective forces on gut microbiomes according to host habitat requirements and environmental niche breadth (Anders et al. 2022; Bouilloud et al. 2024; Heni et al. 2023). However, the diversity and composition of gut microbiomes of sympatric native and invasive host species that may assemble into novel communities in anthropized environments remain poorly understood.

Here, we address the variation in gut microbiome diversity and composition within and among sympatric native and invasive host species that co‐occur across a continuous urban–rural ecotone. For this, we targeted four sympatric small mammal species in Borneo. These species were: one rat endemic to the forest environments of Sundaland (the Mueller's giant Sunda rat *Sundamys muelleri* ) and three key invasive/commensal species that have originated elsewhere (most likely the Indian Peninsular, Aplin et al. 2011; Hutterer and Tranier 1990) and successfully spread in South‐East Asian anthropogenic environments, namely the black rat *Rattus rattus* (species complex), the Norwegian rat *Rattus norvegicus* , and the Asian house shrew *Suncus murinus* . The relatively recent onset of large‐scale logging in Borneo in the 1970's (Gaveau et al. 2014) has led to environmental encroachment that offers a unique opportunity to study possible shifts in species occurrences across ecotones and the potential consequences for species association and biotic interactions (Bordes et al. 2017; Wells et al. 2014; Wilcove et al. 2013). *Sund. muelleri* has been recently described as an urban adapter, preferring pristine natural landscapes but also frequently found in suburban green spaces. The three invasive species, in turn, are considered urban dwellers that differ in their capacity to exploit natural and non‐anthropogenically shaped environments (Blasdell et al. 2022; Wells et al. 2014). We therefore aimed to explore the extent to which host phylogenetic relatedness, land use intensity (LUI), and spatial proximity of host individuals across an ecotone from forest to highly urbanised areas influence gut microbiome composition and diversity in the four sympatric species. We hypothesised that host species identity would play a primary role in shaping the host gut microbiome, with more closely related species having more similar microbiome composition. Among the three rat species, we expected *Sund. muelleri* to exhibit a distinct microbiome compared to the two congeneric invasive *Rattus* species. However, considering the potential influence of the underlying urban–rural ecotone, which represents unique gradients of habitat suitability and varying degrees of overlap in the realised habitat utilisation among host species (Wells et al. 2014), we also anticipated that such overlap in habitat use may influence multi‐host microbiome associations. Moreover, we expected that the urban–rural ecotone would influence shifts in microbiome assemblages among individuals of the same host species.

## 2 Materials and Methods

### 2.1 Study System and Sample Collection

We analysed 245 individual faecal samples of four sympatric small mammals captured on a continuous urban–rural gradient in Kota Kinabalu (lat. 6.0° long. 116.1°), northern Borneo (Sabah, Malaysia), between March 2012 and May 2013 (Wells et al. 2014). The city, which has expanded towards the nearby tropical rainforest in the last 50 years, is surrounded by suburban and village areas interlaced with production forest and nearby old‐growth forest of the Crocker Range biosphere reserve. The captured animals belonged to four species from two families: the native Muller's giant Sunda rat *Sundamys muelleri* , the commensal Asian black rat *Rattus rattus* (species complex), and the commensal Norway rat *Rattus norvegicus* , all three belonging to the tribe Rattini within the family Muridae (Pagès et al. 2016) and the commensal Asian house shrew *Suncus murinus* , from the family Soricidae.

For each trapping location, geographical coordinates and elevation were recorded with a handheld GPS device (Garmin GPSmap62st, Olathe, USA). We recorded the proportional coverage with different landcover types within 20 m radii around trapping locations, categorising and scoring land cover types according to increasing human impact and urbanisation as ‘forest’ = 1, ‘fallow tree’ = 2, ‘fallow grass/agriculture/garden’ = 3, ‘housing soil’ = 4 and ‘housing compound’ = 5 (Wells et al. 2014). The landcover types ‘soil’, ‘sealed’, and ‘water’ were scored as 3 due to the difficulty to categorise their land‐use intensity. We then computed a land use intensity (LUI) by summarising the products of proportional coverage and land cover scores for each trapping location and scaling the resulting vector between 0 and 1.

Captured animals were then transferred to nearby mobile field laboratories for subsequent anaesthesia via diethyl ether inhalation (anaesthetic grade) and then sacrificed by cervical dislocation (according to guidelines by the American Veterinary Medical Association, https://www.avma.org). Species were identified based on morphological characters (Aplin et al. 2003; Carleton and Musser 2005; Musser and Carleton 2005). Faecal samples were collected from dissected colons, stored in ethanol, and frozen at −20°C.

Biological resource access and export permits were issued by the Sabah Biodiversity Centre (JKM‐MBS.1000–2/2[35], JKM‐MBS.1000–2/2[63]); access to forest field sites was approved by Sabah Parks and individual landowners.

### 2.2 DNA Extraction, Library Preparation, and High‐Throughput Sequencing

DNA was extracted from faecal samples (~50 mg) using the prepGEM Bacteria kit (MicroGEM, Charlottesville, VA, USA), according to the manufacturer's instructions, with an additional preliminary wash step using the prepGEM wash buffer to minimise potential surface contamination. Samples were homogenised using bead beating (1.4 mm ceramic beads, 3 × 30 s using a Precellys 24 homogeniser).

We performed a two‐step polymerase chain reaction (PCR) targeting the V4 region of the 16S rRNA gene using the updated sequences of the 515F and 806R primers (Apprill et al. 2015; Parada et al. 2016). The first PCR reaction of 20 μL consisted of 2 μL of DNA, 10 μL of Platinum II Hot‐Start PCR Master Mix (2X) (Thermo Fisher Scientific, Waltham, MA, USA), 0.4 μL of forward and 0.4 μL of reverse primers, and 7.2 μL of ultra‐pure water. Reaction conditions consisted of an initial denaturation at 95°C for 3 min, followed by 28 cycles of 30 s at 95°C, 30 s at 55°C, and 30 s at 72°C, and finally 72°C for 5 min.

During the second PCR, sample‐indexing was performed using the Nextera XT Index Kit (Illumina Inc., San Diego, CA, USA), in a 25 μL reaction consisting of 2.5 μL of amplified DNA from the previous PCR, 12.5 μL of Platinum II Hot‐Start PCR Master Mix (Thermo Fisher Scientific, Waltham, MA, USA), 1.25 μL of each index, and 10 μL of ultra‐pure water. Reaction conditions were as above, but with 8 cycles instead of 28. PCR products were pooled based on agarose gel band relative intensity, cleaned using the AMPure XP beads kit (NEBNext sample purification beads, USA) according to the manufacturer's instructions, and quantified via qPCR using the NEBNext Library Quant Kit for Illumina (Ipswich, MA, USA). Final libraries were normalised to 4 nM and sequenced on an Illumina MiSeq platform (paired 300 bp reads).

### 2.3 Bioinformatics

The raw sequence reads were assigned into amplicon sequence variants (ASVs) using DADA2 (Callahan et al. 2016) within QIIME2 (version 2023.2, Bolyen et al. 2019). After quality checking, raw sequences were truncated at 240 bp (forward) and 220 bp (reverse) and trimmed (leading 19 bp) to avoid potential adaptor contamination, denoised and filtered to remove chimeras. ASVs were taxonomically classified using the naive Bayes classifiers trained on the Silva database (v138, Pruesse et al. 2007) and a phylogenetic tree was constructed using the QIIME2–phylogeny plugin. The ASV taxonomy table and the ASV abundance table generated by QIIME2 were then imported into R version 4.2.3 (R Core Team 2023) for further analysis. ASVs that were not assigned as bacterial sequences were removed, as well as mitochondrial and chloroplast DNA sequences. After inspection of the ASV abundance table, two samples that had 0 and 1 sequences were removed. We then filtered the ASVs to exclude potential artefacts and rare ASVs, retaining only those present in ≥ 5% of samples from any of the species observed to be hosts for the respective ASV. This corresponded to either ≥ 3 samples from *Sund. muelleri*, *R. norvegicus* , or *Sunc. murinus*, or ≥ 4 samples for *R. rattus* . Finally, we excluded individual samples with less than *n* = 4069 reads (minimum library size determined based on acceptable levels of saturation in alpha diversity rarefaction curves). This resulted in a dataset of 236 samples (58 *Sund. muelleri*, 74 *R. rattus* , 49 *R. norvegicus*, 55 *Sunc. murinus*).

Raw sequence reads have been deposited in the European Nucleotide Archive (Accession number PRJEB81284).

### 2.4 Statistical Analyses

To account for heterogeneous sequencing effort (Schloss 2024), we normalised the ASV data by rarefaction, iteratively sampling 100 random subsets of *n* = 4069 reads (according to minimum library size) for each host individual sample, for all statistical analyses based on alpha and beta diversity metrics.

To characterise the microbiome alpha diversity for each host individual, we computed Chao1 richness as an estimate of relative bacterial species richness (Chao 1984) and Shannon entropy as an estimate of relative bacterial species diversity (Shannon 1948). We used Gaussian Generalised Linear Models (GLMs) to explore variation in individual alpha diversity in relation to LUI for each host species (with separate models for each species) and for estimating the expected average individual species richness and diversity (using a single model with all species). Practically, we iteratively computed the alpha diversity metrics (Chao1, Shannon entropy) from the aforementioned normalised subsets, ran the GLMs, and extracted the mean and SE of the intercept and coefficient estimates. We then drew a total of 10,000 ‘posterior’ values from these estimates and the corresponding SEs (from an underlying normal distribution) and reported the mode and 95% credible intervals (CI) as results that account for both the uncertainty arising from the rarefaction and the GLM likelihood procedures. We considered CIs not overlapping zero to represent ‘significant effects’.

We characterised the pairwise similarity in microbial assemblages among host individuals by computing Bray–Curtis (Bray and Curtis 1957) and weighted UniFrac (Lozupone and Knight 2005) beta diversity metrics. We then used a Generalised Dissimilarity Model (GDM) as a matrix‐based regression approach (Ferrier et al. 2007; Mokany et al. 2022) to explore changes in these beta diversity metrics. As explanatory variables, we included host phylogenetic relatedness, computed as the pairwise phylogenetic distance between host individuals based on a consensus phylogenetic tree generated from the vertlife.org project, accessed in June 2023 (Upham et al. 2019); LUI, which was transformed into dissimilarity metrics during model fitting; and spatial proximity, calculated as the geodesic distance between trapping locations. GDM uses non‐linear splines to describe how changes in predictor variables (i.e., host phylogenetic relatedness, LUI, spatial proximity) influence similarity (i.e., Bray–Curtis and weighted UniFrac) between samples. Since the utilised GDMs with an underlying negative exponential link function assume a monotonic increase in dissimilarity in relation to predictor variables, the height of the splines indicates the relative importance of the predictor variable (i.e., the total amount of compositional variation associated with the predictor variable being evaluated), while its shape indicates the rate of change in the beta diversity measures across the predictor's values, with a steeper slope indicating higher compositional variation at a given data point (Mokany et al. 2022). GDMs were performed using the R package ‘gdm’ (Fitzpatrick et al. 2022), whereby we ran models considering (*gdm1*) all host individuals together, (*gdm2*) the three rat species only ( *Sund. muelleri* , *R. rattus* , *R. norvegicus* ), and (*gdm3*) each rat species separately. Practically, we iteratively calculated Bray–Curtis and weighted UniFrac diversity metrics for the 100 subsets, ran the GDMs, extracted the partition of deviance estimates, and calculated the ‘posterior’ mode and 95% CI from the resulting distribution of estimates. From each GDM, we also extracted the estimated spline for each predictor variable.

We performed analysis of similarity (ANOSIM) across all host species and specific pairwise combinations to investigate the similarity in microbiome composition among the studied species, using the function ‘anosim’ from the package ‘vegan’ (Martinez 2020). We investigated beta diversity dispersion of microbial assemblages in different host species (homogeneity/variance of distance‐to‐centroid dispersion for each host species) using the function ‘betadisper’ from the package ‘vegan’ (Dixon 2003), and the Tukey honestly significant difference (HSD) to test for pairwise differences in the beta dispersion among host species. In addition, we tested the relationship between beta diversity dispersion (distance‐to‐centroid scores) and LUI separately for each host species using Gaussian GLM.

To visualise the variation in microbiome composition within and among host species, we used non‐metric multidimensional scaling (NMDS) based on averaged Bray–Curtis and weighted UniFrac measures.

Finally, we performed ASV differential abundance analysis using Analysis of Composition of Microbiomes with Bias Corrections 2 (ANCOM‐bc2) (Lin and Peddada 2020, 2024) to investigate how shifts in microbial composition allow us to trace changes in the relative abundance of ASVs in relation to the LUI within each host species. This method includes *p*‐value adjustments to control for multiple testing and reduce the false discovery rate, as well as a sensitivity analysis to minimise false positives due to the pseudo‐counts added to handle zero values. We performed ANCOM‐bc2 separately for each host species for all ASVs with > 10% prevalence in the respective species. ASVs were defined as significantly differentially abundant if their adjusted *p*‐value was < 0.05.

### 2.5 Occurrence of Host Species Across the Urban–Rural Ecotone

**FIGURE 1:**
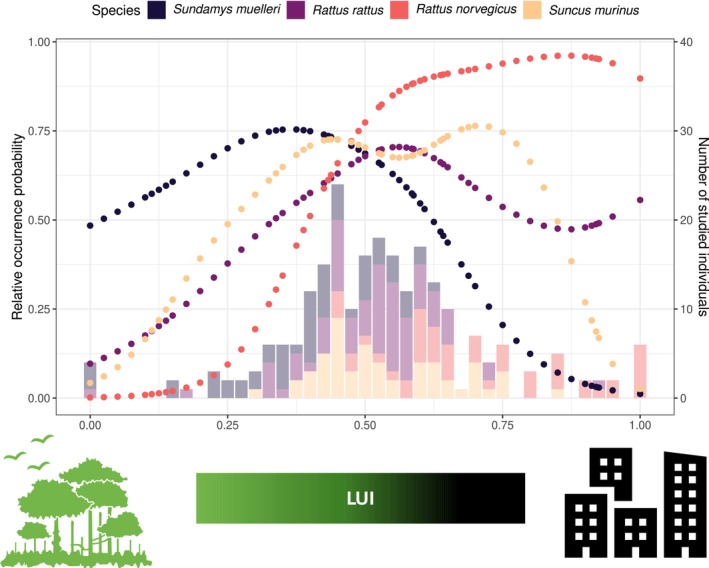
Illustration of the occurrence/co‐occurrence patterns of the four focal small mammal species across the urban–rural gradient in Borneo. Dotted lines show the estimated relative occurrence probabilities of species across the underlying gradient of land use intensity (LUI), stacked bars show the number of individuals for which microbiomes have been analysed in this study matched to assigned LUI values. Bar colours match the colours of the dotted lines but are displayed with increased transparency.

In order to generate an index of habitat suitability based on whether a species is more likely to occur in a location with a certain land use intensity than others across the urban–rural ecotone, we quantified the relative occurrence probability of each focal species along the urban–rural gradient based on capture success; for this we regressed for each species the presence‐absence records for 3538 trap locations (from Wells et al. 2014) against the LUI metric and with a spatial smoothing term of paired geographical coordinates (in order to account for spatial autocorrelation) in a binomial generalised additive models (GAM) using the package ‘mgcv’ (Wood 2017). The results of this analysis showed that the three rat species occurred in distinct pattern across the gradient of LUI, with *Sund. muelleri* predominantly present in more natural environments characterised by low to moderate LUI, *R. rattus* occurring along the entire gradient but most frequently in areas of moderate LUI, and *R. norvegicus* mostly found in semi‐urban to strongly urbanised areas, characterised by average to high LUI. The shrew *Sunc. murinus* occurred most often in areas with moderate to high LUI, while it was unlikely to occur in natural or strongly urbanised areas. In summary—as outlined in our previous work (Wells et al. 2014) – the four sympatric species exhibited clearly distinct habitat preferences, while also exhibiting some overlap in habitat use and occurring in close proximity to each other (Figure 1). Since metrics of individual habitat suitability and associated LUI values were correlated for *R. norvegicus* and *Sund. muelleri*, we only considered LUI in the analyses.

## 3 Results

### 3.1 Faecal Microbiome Composition

A total of 1864 unique bacterial ASVs were identified after quality checking ( *Sundamys muelleri* = 1294 ASVs, *Rattus rattus* = 1261 ASVs, *Rattus norvegicus* = 850 ASVs, *Suncus murinus* = 246 ASVs; Figure S1), of which 64 were found in all four host species and none was found in all 236 individuals. At the species level, *Sund. muelleri* had 990 ASVs in common with *R. rattus* (> 76% of the number of ASVs found in any of these two species) and 491 ASVs in common with *R. norvegicus* , while 577 ASVs were found in both focal *Rattus* species (46% of ASVs found in *R. rattus* and 68% of ASVs found in *R. norvegicus* ). The shrew *Sunc. murinus* had 111 ASVs in common with *Sund. muelleri*, 120 ASVs with *R. rattus* , and 118 ASVs with *R. norvegicus* .

**FIGURE 2:**
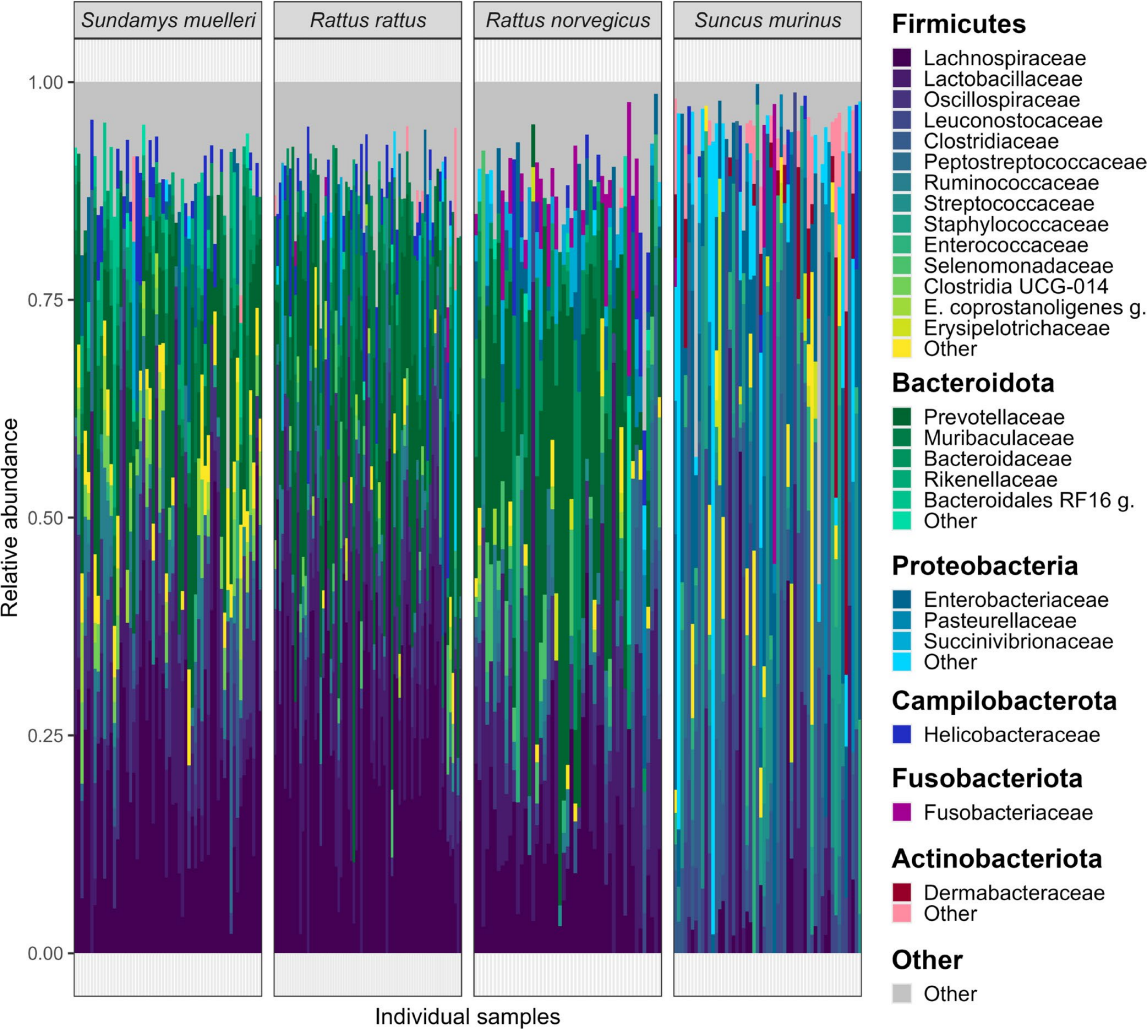
Compositional plot of the bacterial families found in the faecal microbiomes of host individuals from four small mammal species studied in Borneo. For each individual, families representing less than 2% relative abundance were clustered together as ‘Other’. ( *E. coprostanoligenes* g.: *Eubacterium coprostanoligenes* group; Bacteroidales RF16 g.: Bacteroidales RF16 group.).

The three most abundant bacterial families in all three rat species were *Lachnospiraceae*, *Prevotellaceae*, and *Lactobacillaceae*, with the first family being the most abundant in *R. rattus* and *Sund. muelleri*, and the second being most abundant in *R. norvegicus* , respectively (Figure 2; Figure S2). The family *Muribaculaceae* was mainly detected in *R. rattus* and *Sund. muelleri*, while the families *Peptostreptococcaceae* and *Enterobacteriaceae* were mostly found in *R. norvegicus* and *Sund. muelleri*. Of the less frequent bacterial families, *Selenomonadaceae*, *Fusobacteriaceae*, and *Succinivibrionaceae* were mostly detected in *R. norvegicus* , while *Rikenellaceae* and the taxa *Clostridia UCG‐014* were mainly found in *Sund. muelleri*. The faecal microbiome composition of the shrew *Sunc. murinus* was clearly distinct from those of the three rat species, with the most abundant families *Clostridiaceae* and *Leuconostocaceae* of the shrew microbiome being rarely found in the rat species. The less frequent families *Dermabacteraceae, Erysipelotrichaceae, Staphyloccoccaceae*, and *Pasteurellaceae* were primarily detected in *Sunc. murinus*, while they were nearly absent (with relative abundances < 1% or undetectable) in the rat species. These clear differences in microbiome composition of the rat species and *Sunc. murinus* at the family level also translated into distinct microbiomes at the phylum level, with *Bacteroidota* predominantly found in the rat species and *Proteobacteria* most abundant in *Sunc. murinus* (Figure 2; Figure S2). *Sunc. murinus* also exhibited higher species‐specific variance in microbiome composition compared to the three rat species, with lower consistency in individual microbiome composition (Figure 2).

### 3.2 Faecal Microbiota Alpha and Beta Diversity

The average individual‐level richness estimates of microbial assemblages were highest in *Sund. muelleri* (Chao1: 129, 95% CI of 119–137) and *R. rattus* (Chao1: 142, 95% CI of 133–150), slightly lower in *R. norvegicus* (Chao1: 103, 95% CI of 93–114) and lowest in the shrew *Sunc. murinus* (Chao1: 33, 95% CI of 24–43) (Figure S3; Table S1). Likewise, we found the highest average Shannon diversity in *Sund. muelleri* and *R. rattus* and the lowest in *Sunc. murinus* (Figure S3; Table S1). We found no evidence that variation in alpha diversity among individuals from any of the host species correlated with land use intensity (LUI) (according to zero‐overlapping CIs from GLMs; Figure S3; Table S1).

**FIGURE 3:**
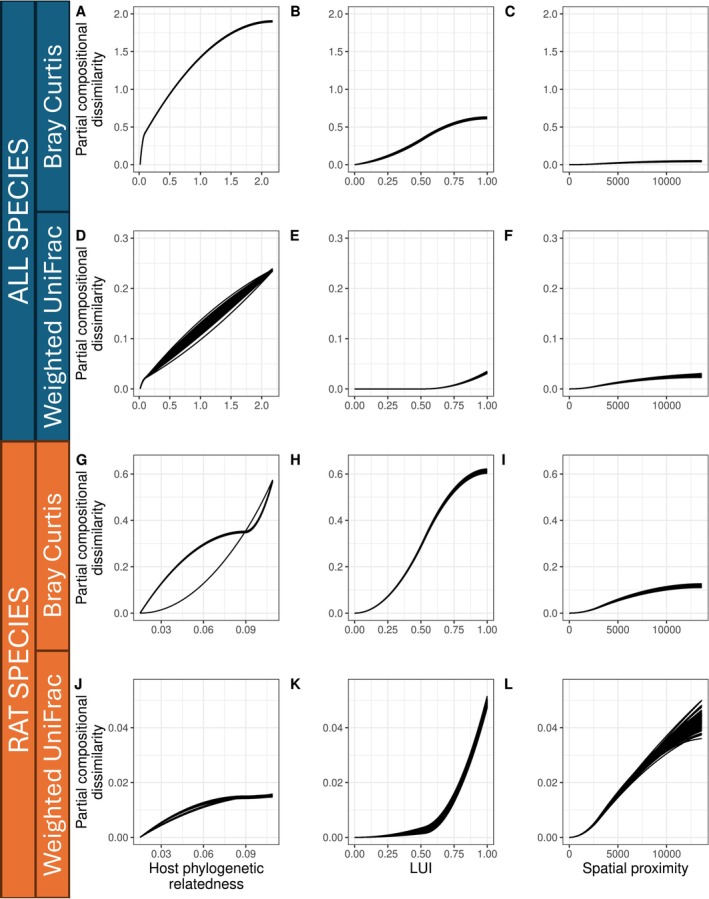
Relative influence of host phylogenetic relatedness, land use intensity (LUI) and spatial proximity on microbial community variation within and among individuals from all four small mammal species (A–C for Bray–Curtis and D–F for weighted UniFrac) and the three rat species only ( *Sundamys muelleri* , *Rattus rattus* , *Rattus norvegicus* ) (G–I for Bray–Curtis and J–L for weighted UniFrac). The effects are plotted as fitted I‐splines from Generalised Dissimilarity Models exploring the relationship between Bray–Curtis and weighted UniFrac beta diversity as response variables and the three predictor variables (host phylogenetic relatedness, LUI, spatial proximity). Spline height indicates the beta diversity variation explained by each predictor, while slope indicates the rate of change in microbiome assemblage along each predictor's range. Splines are plotted for iterative model fitting to rarified data subsets (see methods).

Exploring the variation in microbial assemblage composition within and among all host species, assemblage similarity was mostly explained by host phylogenetic relatedness (> 30% of deviance explained for both Bray–Curtis and weighted UniFrac metrics in all‐species GDMs (*gdm1*)) (Figure 3; Table 1). In *gdm1*, LUI explained only a small fraction of the deviance in assemblage similarity for both Bray–Curtis (1.44%) and weighted UniFrac (0.29%) metrics as response variables. In these models, the variation of Bray–Curtis was mostly evenly distributed along the LUI gradient with some minor variation and smaller LUI values and a plateau towards large LUI values in highly urbanised environments, whereas variation in weighted UniFrac in microbial assemblages was mostly associated with relatively high LUI values according to the fitted functional splines (Figure 3; Table 1). This suggests that microbial community variation associated with higher LUI in more urbanised environments exhibits the most pronounced phylogenetic compositional variation as captured by the weighted UniFrac metric.

**TABLE 1:** Deviance explained by host phylogenetic relatedness, land use intensity (LUI), and spatial proximity for Bray–Curtis and weighted UniFrac beta diversity calculated by generalised dissimilarity models fitted to all species (on the left) and rat species only (on the right). Values are reported as mode [95% CI].

Predictor variable	All species		Rat species	
	Bray–Curtis	Weighted Unifrac	Bray–Curtis	Weighted Unifrac
Host phylogenetic relatedness	34.65 [34.45–34.80]	31.28 [31.03–31.45]	9.33 [9.22–9.47]	0.38 [0.36–0.39]
LUI	1.44 [1.40–1.47]	0.29 [0.26–0.32]	2.70 [2.62–2.77]	1.13 [0.99–1.19]
Spatial proximity	0	0	0.18 [0.16–0.20]	0.64 [0.58–0.69]
Host phylogenetic relatedness ꓵ LUI	0	0	1.35 [1.33–1.37]	0
Host phylogenetic relatedness ꓵ Spatial proximity	0	0	0.06 [0.06–0.06]	0
LUI ꓵ Spatial proximity	0	0	0.06 [0.06–0.06]	0.21 [0.20–0.22]
Host phylogenetic relatedness ꓵ LUI ꓵ Spatial proximity	0	0	0.06 [0.06–0.06]	0
Unexplained	64.58 [64.44–64.79]	68.71 [68.55–69.00]	86.28 [86.10–86.37]	97.74 [97.63–97.85]

In the GDMs for the three rat species only (*gdm2*), the overall deviance explained by covariates was lower compared to *gdm1* (14% for Bray–Curtis and 2% for weighted UniFrac). However, in these models, the total amount of compositional variation in microbiome assemblages associated with LUI for Bray–Curtis and weighted UniFrac (Figure 3H,K) and spatial proximity for weighted UniFrac (Figure 3L) was of similar or even higher relative importance than compositional variation explained by host phylogenetic relatedness (Figure 3, Table 1). In fact, the LUI explained more deviance in *gdm2* compared to *gdm1* models (2.70% vs. 1.44% for Bray–Curtis and 1.13% vs. 0.29% for weighted UniFrac). The functional relationships between LUI and compositional variation in GDM models for rat species only (*gdm2*) were similar to those in models for all species (*gdm1*) in that the variation of Bray–Curtis was mostly constant along the LUI gradient with a plateau towards large LUI values and variation in weighted UniFrac mostly associated with relatively high LUI values (Figure 3).

Species‐specific GDMs (*gdm3*) revealed a notable impact of land use contrast on assemblage similarity in *R. norvegicus* individuals only (7% and 3.5% of deviance explained for Bray–Curtis and weighted UniFrac as response variables; Table S2).

**FIGURE 4:**
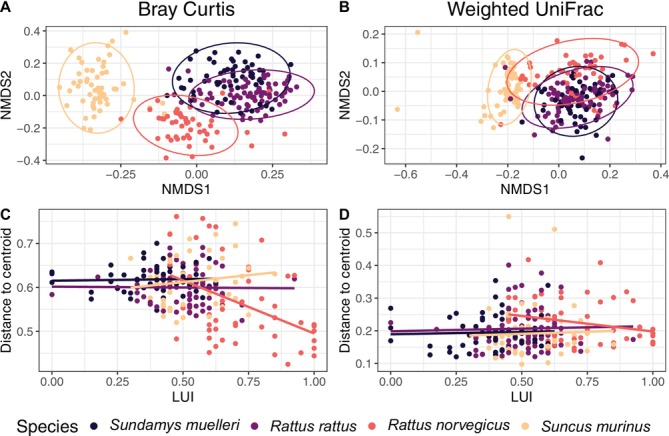
(A, B) Non‐metric multidimensional scaling (NMDS) plots of the faecal microbiome of the four studied species based on Bray–Curtis (A) and weighted UniFrac (B) dissimilarity metrics. Ellipses represent 95% confidence intervals. (C, D) Scattered plot of individual sample distance to host species centroid based on Bray–Curtis (C) and weighted UniFrac (D) plotted against underlying land use intensity (LUI) (0 = natural environment, 1 = urbanised environment); linear regression lines are shown for each host species. (Sample sizes: *n* = 58 for *Sund. muelleri*, *n* = 74 for *R. rattus* , *n* = 49 for *R. norvegicus* , *n* = 55 for *Sunc. murinus*).

While analysis of similarity (ANOSIM) and multidimensional scaling (NMDS) visualisation (Figure 4A,B) confirmed that microbial assemblage composition was most similar for host individuals from the same species for both Bray–Curtis and weighted UniFrac measures (Bray–Curtis: ANOSIM: *R* value = 0.70, *p* < 0.001; weighted UniFrac: ANOSIM: *R* value = 0.49, *p* < 0.001), there was a clear overlap in the assemblage compositions in *Sund. muelleri* and *R. rattus* individuals and, to a lesser extent, also in assemblages from other pairs of host species. The similarity in terms of microbiome composition between these two species was corroborated by the pairwise ANOSIM, which showed the smallest R value between *Sund. muelleri* and *R. rattus* compared to the other host species pairs (Table S3). For both beta diversity measures, *Sunc. murinus* microbiome assemblages were clearly distinct from the three rat species, with *R. norvegicus* as the closest rat species in both cases according to ANOSIM results (Figure 4A,B; Table S3).

The beta diversity dispersion of microbial assemblages for Bray–Curtis revealed significantly lower average distance from the centroid in *R. norvegicus* compared to the other three host species (PERMDISP: df: 3, *F* value: 10.14, *p*: 0.001; Figure S4; Table S4). However, the beta diversity dispersion of *R. norvegicus* was also characterised by a number of individuals dispersing most strongly from the centroid compared to more homogeneously distributed assemblage dispersion among individuals of the other host species (Figure S4). This dispersion (Bray–Curtis assemblages of *R. norvegicus* ) decreased with increasing LUI (GLM on PERMDISP distance to centroid and LUI, *β* = −0.24, SE = 0.07, *p* < 0.01), suggesting that host individuals of *R. norvegicus* in less urbanised environments harboured the most distinct microbial assemblages (Figure 4C; Table S5). These patterns were not observed for weighted UniFrac, as we found no association between the dispersion from centroid and LUI and the weighted UniFrac dispersion of assemblage compositions in *R. norvegicus* was not significantly different from that measured for assemblages in the other three host species for this metric (Figure 4D; Table S5).

### 3.3 Shifts in ASV Relative Abundances Across the Urban–Rural Ecotone

LUI significantly influenced the relative abundance of 83 ASVs (out of 946 ASVs with a species‐specific prevalence > 10%) in the four small mammal species, with the ASVs assigned to 30 different bacterial families (ANCOM‐bc2, *adj p* < 0.05; Table S6). The largest number of differentially abundant ASVs in association with LUI was found in *R. norvegicus* with 73 ASVs, compared to 8 ASVs in *Sunc. murinus* and 3 in *Sund. muelleri*. We found no evidence of such ASV variations in *R. rattus* .

**FIGURE 5:**
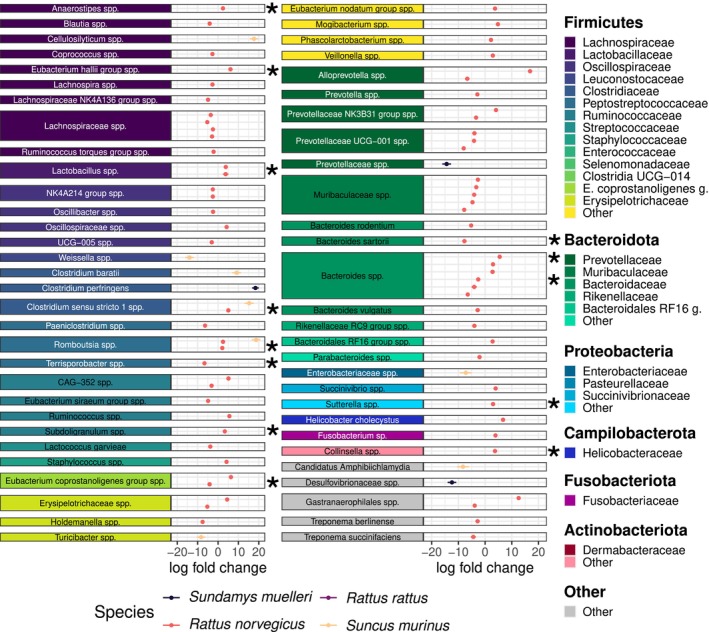
Differentially abundant ASVs in relation to land use intensity (LUI) found in the four sympatric small mammal species. The log fold change represents the effect size of a one‐unit increase in LUI on the abundance of the ASV as determined by compositional analysis with bias correction (ANCOM‐bc2). ASVs are grouped and coloured by taxonomic assignment. Points represent the average log fold change and bars the 95% CI. Asterisks identify the differentially abundant ASVs that passed the sensitivity test for pseudo‐count addition. ( *E. coprostanoligenes* g.: *Eubacterium coprostanoligenes* group; Bacteroidales RF16 g.: Bacteroidales RF16 group.).

In *R. norvegicus* , the highest number of differentially abundant ASVs was reported for the bacterial families *Lachnospiraceae* (11 ASVs), *Bacteroidaceae* (9 ASVs), and *Prevotellaceae* (8 ASVs). Out of these 73 differentially abundant ASVs, 43 (59%) exhibited a decrease in their relative abundance with higher LUI (ANCOM‐bc2, *adj p* < 0.05; Table S6). Most of these ASVs were from the families *Lachnospiraceae* (9/11), Oscillospiraceae (4/5), *Prevotellaceae* (6/8), *Muribaculaceae* (5/5), and *Bacteroidaceae* (6/9) (Figure 5). In *Sund. muelleri*, *Clostridium perfringens* was reported to be higher in abundance in more urban environments, while the other two differentially abundant ASVs found in this species were significantly less abundant with higher LUI (ANCOM‐bc2, *adj p* < 0.05; Figure 5; Table S6). In *Sunc. murinus*, *Clostridium baratii* and three other ASVs were more abundant in more urban environments, while 4 ASVs decreased in their relative abundance with higher LUI (ANCOM‐bc2, *adj p* < 0.05; Figure 5; Table S6). *Romboutsia* spp. was the only ASV found to be differentially abundant in two different host species, *R. norvegicus* and *Sunc. murinus*, reflecting in both cases a positive effect of LUI.

Of these 83 differentially abundant ASVs, 13 passed the sensitivity test for the pseudo‐count addition, all belonging to *R. norvegicus* : *Anaerostipes* spp., *Eubacterium halii group* spp., *Lactobacillus* spp., *Clostridium sensu stricto 1* spp., *Romboutsia* spp., *Subdoligranulum* spp., *Bacteroides* spp., *Sutterella* spp., and *Collinsella* spp. were positively associated with LUI (higher abundance with higher LUI), whereas *Terrisporobacter* spp., *Eubacterium coprostanoligenes group* spp., *Bacteroides sartorii* , and *Bacteroides* spp. were negatively associated with LUI (lower abundance with higher LUI) (ANCOM‐bc2, *adj p* < 0.05, ss = TRUE; Table S6).

## 4 Discussion

Understanding the patterns and processes that shape the microbial communities associated with animal species that exploit modified and urbanised landscapes could fill some important knowledge gaps of how native species are resilient to habitat changes and commensal species encroach into modified habitats.

Here, we explore the gut microbiome composition of four sympatric small mammal species across an urban–rural ecotone in Borneo and show that despite distinct microbiomes among all host species, the native forest rat *Sundamys muelleri* , which occupies both natural and suburban habitats as an urban adapter, and the commensal rat *Rattus rattus* , which is an urban dweller but capable of exploiting suburban and semi‐natural environments, exhibited the highest similarity in microbiota. We further show that the relative abundance of specific bacterial taxa correlates with land use intensity (LUI), whereby the strongest changes in microbiota in relation to LUI were found in the urban‐dwelling commensal rat *Rattus norvegicus* , which has limited capacity to explore suburban and rural areas of moderate to low land use intensity in our study area. Our findings reveal a strong phylogenetic signal when comparing individuals from distinct host species, while underlying habitat conditions play an important role among closely related species. These results provide some first evidence that ecological opportunity, possibly arising from the co‐occurrence and environmental niche overlap of native and invasive species, may facilitate similarities in gut microbiomes, amid environmental forcing as reflected in the impact of land use intensity on specific ASV abundances.

### 4.1 Host Biological Features and Niche Overlap Correlate With Host‐Microbiome Associations

Host species identity was the strongest factor shaping the microbiome of the four studied small mammal species. In particular, we found that the bacterial community of the shrew *Suncus murinus* was clearly distinct from those of the three rat species (*Sund. muelleri*, *R. rattus* , and *R. norvegicus* ), but among these latter species, we found the strongest similarity in microbial communities between *Sund. muelleri* and *R. rattus* rather than the two congeneric *Rattus* species. Our results, therefore, contrast with the commonly found pattern of closely related mammalian species harbouring the most similar gut microbiomes (Amato et al. 2019; Heni et al. 2023; Kartzinel et al. 2019), while they are consistent with those of other studies on small mammals that reported the strongest effects of host phylogeny on microbiome composition when comparing distantly related host species and much weaker effects near the tips of phylogenies (Brown et al. 2023; Knowles et al. 2019). Phylogeny impacts the host microbiome by shaping host physiology, including immune system function and gut morphophysiology. These factors influence microbial selection and diversification, which are further modulated by diet, as well as vertical and horizontal transmission of microbes (Mallott and Amato 2021; Maritan et al. 2024). The clearly distinct faecal microbiota between the studied shrew and rats as well as the lower species richness in shrew matched our expectations with regards to taxonomy, but also diet and host gut morphology. The shrew *Sunc. murinus* is an insectivorous species, typically having a shorter and simpler gastrointestinal tract compared to omnivorous species (Boonzaier et al. 2013; Shinohara et al. 2019). Differences between the gut microbiome of insectivorous versus omnivorous mammals have been scarcely investigated in comparative studies to date. Lower microbiome diversity in insectivores compared to sympatric omnivores has been also observed in primates from the family *Strepsirrhines* (Bornbusch et al. 2019, 2022) and in the insectivore shrew *Crocidura russula* compared to the omnivore mouse *Apodemus sylvaticus* (Koziol et al. 2023), whereas such differences were not found among sympatric desert rodents (Kohl et al. 2022). More generally, if we consider insectivores as specialised towards a narrower diet range than omnivorous rodents, our results align with studies showing that carnivores have less diverse microbiomes compared to omnivores and herbivores (Ley et al. 2008; Zoelzer et al. 2021). The bacterial families *Lachnospiraceae*, *Prevotellaceae*, and *Bacteroidaceae*, which were dominant in the rats in this study, were not only reported to dominate in other rodents (Gu et al. 2013) but also to comprise core microbial members of large hindgut fermenters (O' Donnell et al. 2017). On the other hand, the shrew's dominant symbiotic bacterial families *Clostridiaceae*, *Enterobacteriaceae*, and *Peptostreptococcaceae* were reported as more prevalent in carnivores compared to omnivores and herbivores (de Jonge et al. 2022; Zoelzer et al. 2021).

Given the limited influence of phylogeny on rat species' microbiomes and the strong impact of diet on microbiome composition observed in many comparative studies (Kartzinel et al. 2019; Ley et al. 2008), it would be of interest to analyse how the diet of our focal rat species varied among species and also the urban–rural ecotone. Unfortunately, we currently lack details about the diet and dietary variation of these three rat species. As omnivore species, one can expect some opportunistic feeding on different food items for all three rat species, while across urban‐suburban gradients, available food sources likely shift from more natural products to human‐derived processed food and organic waste items, including those from local restaurants and markets. Alternatively, the exchange of microbes with the environment is another pathway through which gut microorganisms can colonise hosts, and overlaps of habitat use among host species have been associated with more similar gut microbiomes in humans and other mammals (Knowles et al. 2019; Raulo et al. 2024; Rothschild et al. 2018; Teng et al. 2022). Moreover, social interactions, both within and between host species, can further enhance microbial dispersal between individuals, influencing host‐associated microbiome composition and increasing or decreasing microbiome similarity among more or less connected host individuals (Raulo et al. 2024; Sarkar et al. 2020; Stothart et al. 2021). Across the studied urban–rural ecotone, *Sund. muelleri* and *R. rattus* were both found to exploit habitats of intermediate land use intensity, which corresponded to suburban vegetation patches with some tree or shrub cover and nearby housing. In Borneo and elsewhere within its geographic range, *Sund. muelleri* is frequently recorded in pristine and logged forest environments (Wells et al. 2007), while it was only recently found to also thrive in suburban vegetation as an urban adaptor (Blasdell et al. 2022; Wells et al. 2014). *R. rattus* , as a commensal species, in turn, is highly adapted to urban conditions where it potentially also overlaps in habitat use with *R. norvegicus* ; however, it is also capable of exploiting natural vegetation and even forests near human infrastructures (Loveridge et al. 2016; Wells et al. 2006). *R. norvegicus*, on the other hand, was predominantly reported in urban areas or suburban vegetated patches near streams or sewage and urban infrastructure in our study area (Wells et al. 2014). Elsewhere, *R. norvegicus* has also been reported to benefit from sewage systems or habitat and soil conditions that allow excavating sufficiently large burrowing systems for their colonies (Feng and Himsworth 2014), suggesting that habitat features linked to sheltering colonies might be a constraining factor in exploiting novel environments as much as diet.

Apparently, with more similar microbiomes found in the more distantly related rat species *Sund*. *muelleri* and *R. rattus* , strong co‐evolution and co‐speciation among hosts and associated microbiota cannot be the sole driving force at work (Groussin et al. 2020; Mazel et al. 2023). Our findings suggest that host biological features and some aspects of niche overlap (perhaps diet sharing or environmental exposure) may synergistically drive the observed patterns of microbiome sharing.

### 4.2 Land Use Intensity Influences the Microbiome Most Strongly in *Rattus norvegicus*

Exploring changes in the microbiome across a continuous land use gradient from urban to forest habitats, we found some evidence that land use intensity (LUI) was associated with changes in microbiome composition and ASV relative abundance in the analysed small mammal species.

Surprisingly, despite *R. norvegicus* being an urban dweller with an almost worldwide distribution, its microbiome composition revealed the strongest relationship with land use intensity, with relatively homogeneous microbiome composition in individuals exploiting urban environments and more distinct (dispersed from the ‘average’ according to Bray–Curtis compositional variation) microbiomes in those individuals exploiting less urbanised environments. The underlying food landscape that might be of relevance for *R. norvegicus* in our study area is complex and currently incompletely understood. The urban environments where this species was trapped include regional food markets as well as nearby restaurants and urban canalisation and drainage. Since this rat species is unlikely to occur in natural habitats in Borneo, we expect that individuals in less urbanised environments will still exploit similar food items, though in reduced quantities due to fewer food vendors and housing compounds. Additionally, they may need to increase their intake of food sourced from nature resulting in a more heterogeneous diet. Such scenario could explain the stronger divergence in microbiome composition of the individuals captured in less urbanised areas and the more homogenised microbiomes in individuals captured in the most urbanised areas. More homogeneous microbiomes in more urbanised areas were also found in bird nests (Maraci et al. 2022), the skin microbiome of amphibians (Zhou et al. 2023) and even urban soil microbial community (Delgado‐Baquerizo et al. 2021) suggesting that urban homogenisation of microbial communities is a common phenomenon.

*R. norvegicus* also displayed the highest number of differentially abundant ASVs in relation to LUI. In particular, the relative abundance of various ASVs from the phylum *Bacteroidota*, particularly the family *Prevotellaceae*, and the family *Lachnospiraceae* was significantly decreased in individuals captured in more urban environments. These fibre degrading taxa have previously been reported as significantly less abundant in humans and mice consuming high‐fat low‐fibre diets (Bailén et al. 2020; De Filippo et al. 2010; Pasolli et al. 2019; Velázquez et al. 2019) and in non‐human primates exposed to anthropogenic activities and human food waste (Moy et al. 2023; Wasimuddin et al. 2022). Additionally, we found the relative abundance of *Treponema berlinense* and *Treponema succinifaciens* from the family *Spirochetaceae* to be significantly depleted in individuals trapped in urban areas. While taxa from this family are generally assumed to be reduced in their abundance in industrialised human populations (De Filippo et al. 2010; Pasolli et al. 2019), the two *Treponema* species we found in commensal *R. norvegicus* have been previously reported in non‐industrialised, rural (Bedouin) human populations in Arabia (Angelakis et al. 2016) and several non‐human primates not exposed to urban environments (Manara et al. 2019). While these results may be ‘snapshot observations’ and warrant future research to explore in more depth microbiome associations in response to dietary changes across urban–rural gradients, they lead to the hypothesis that in less urbanised areas, *R. norvegicus* may need to rely on different, perhaps more natural food resources compared to those in urban environments and such dietary shift are associated with changes in gut microbiomes. Whether the alteration of *R. norvegicus* gut microbiome in less urbanised environment would compromise host health or is linked to demographic features remain unknown. An interesting avenue of research could be more detailed comparisons of microbiome changes in *R. norvegicus* and *R. rattus* at larger biogeographical scale, given their joint and worldwide occurrence in urban environments but differing capacities to exploit natural habitats. Notably, microbial abundance in *R. rattus* was not correlated with LUI in this study, being the only small mammal species without observed differentially abundant ASVs.

Contrarily to our expectations, we found little evidence that the microbiome composition and diversity of the native species *Sund. muelleri* are strongly affected by land use change across the studied ecotone. As an urban adapter species that appears to have expanded its habitat range from forests to sub‐urban woody vegetation and garden areas with the onset of rainforest logging in Borneo (Blasdell et al. 2022; Wells et al. 2014), we would have expected it to encounter novel environmental conditions across the studied ecotone that have been mostly absent in the original natural habitat of this species.

Although we did not find a strong effect of LUI on the microbiome of *Sund. muelleri*, it is worth noting that *Clostridium perfringens* , a well‐known potential pathogen for humans and other animals (Kiu and Hall 2018), was significantly more abundant in individuals found in more urban environments. This bacterium is commonly found in the urban environment as well as in the microbiota of humans and other vertebrates (Kiu and Hall 2018); therefore, its higher abundance in rats captured in more urbanised areas is not necessarily a direct threat for people and other animals. However, in the context of our findings of considerable microbial similarity between *Sund. muelleri and R. rattus*, which is a well‐known vector of zoonotic pathogens (Blasdell et al. 2022; Kosoy et al. 2015; Panti‐May et al. 2017), the detection of a zoonotic bacterium species in *Sund. muelleri* leads to (mostly unanswered) questions around whether native‐invasive small mammal species interfaces could facilitate the host shifting and spillover of parasites from wildlife to humans and vice versa, and whether urban‐adapted species like *Sund. muelleri* could amplify the risk of zoonotic disease outbreaks (Roberts et al. 2021).

## 5 Conclusion

Our study uncovers multi‐host microbiome associations across a continuous urban–rural ecotone that should motivate future research into how such patterns and the possible underlying mechanisms play a role in the constraints of how native and invasive species exploit habitats in times of global change.

We highlight how host phylogeny shapes microbiome differences between distantly related host species, while environmental factors such as co‐occurrence and niche overlap across land use gradients are likely to converge microbiomes among closely related and sympatric species. We suggest that the ecological niche overlap between the native urban adapter *Sundamys muelleri* and the invasive *R. rattus* offers ecological opportunity that shapes microbial associations, while even for generalist host species such as the cosmopolitan *R. norvegicus* , changes in environmental conditions encountered across fine‐scale land use gradients can drive variation in microbiome composition and bacterial abundance. We found first evidence that such shifts in microbial associations across urban–rural ecotones can be of relevance for the abundance of zoonotic gastrointestinal pathogens associated with rodents, but future research is necessary to understand how shifts in microbial associations across land use gradients are linked to changes in habitat suitability, diet, and altered host health and whether plasticity and selective forces on gut microbiomes could limit the way native and invasive species are able to exploit novel environments.

## Author Contributions

**A.G.:** designed research (equal); performed molecular analysis and bioinformatics (equal); performed formal data analysis (lead); wrote the paper – original draft (lead), review and editing (equal). **M.B.L.:** performed field work (support); wrote the paper – review and editing (equal). **F.Y.Y.T.:** performed field work (support); wrote the paper – review and editing (equal). **M.H.:** performed molecular analysis (equal); wrote the paper – review and editing (equal). **S.C.:** wrote the paper – review and editing (equal). **T.U.W.:** designed research (equal); supervised the work (equal); supervised molecular analysis and bioinformatics; wrote the paper – review and editing (equal). **K.W.:** designed research (equal); supervised the work (equal); performed field work (lead); supervised formal analysis; wrote the paper – review and editing (equal).

## Disclosure

Benefits Generated: This research involved the local National Parks authorities (Sabah Parks), with the research motivated to collaboratively study the potential interaction of native and invasive small mammal species found in Borneo's rainforests and anthropogenic environments. Benefits from this research accrue from findings, the sharing of our data and results in public databases, and institutional capacity building.

## Ethics Statement

Biological resource access and export permits were issued by the Sabah Biodiversity Centre (JKM‐MBS.1000–2/2[35], JKM‐MBS.1000–2/2[63]); access to forest field sites was approved by Sabah Parks and individual landowners. No ethical approval (other than the access permit) was required for the capture of the animals involved in this study.

## Conflicts of Interest

The authors declare no conflicts of interest.

## Supporting information


Data S1.

## Data Availability

The dataset (raw sequence reads) analysed during the current study is available in the European Nucleotide Archive (Accession number PRJEB81284). R code for data analysis and display are available from the Zenodo repository: https://doi.org/10.5281/zenodo.15223040.
