# Supplementary material for: Host‐Microbiome Associations of Native and Invasive Small Mammals Across a Tropical Urban–Rural Ecotone
Source: Mol Ecol. 2025 Apr 28;34(11):e17782. doi: 10.1111/mec.17782 (PMC12100590; doi:10.1111/mec.17782)
Supplement: Supplementary file 1 — Data S1. [file MEC-34-e17782-s001.pdf]

MOLECULAR ECOLOGY

Supplemental Information for:

Host-microbiome associations of native and invasive small mammals across a tropical urban-rural ecotone

Alessandra Giacomini[1] (a.giacomini.2156511@swansea.ac.uk), Maklarin B. Lakim[2] (maklarinlakim@gmail.com), Fred Y. Y. Tuh[2] (fredtuh@gmail.com), Matthew Hitchings[3] (m.hitchings@swansea.ac.uk), Sofia Consuegra[1,4] (s.consuegra@swansea.ac.uk), Tamsyn Uren Webster[1] (t.m.urenwebster@swansea.ac.uk), Konstans Wells[1] (k.l.wells@swansea.ac.uk)

Table of Contents:

**Figure S1**	Page 2
**Figure S2**	Page 3
**Figure S3**	Page 4
**Figure S4**	Page 5
**Table S1**	Page 6
**Table S2**	Page 7
**Table S3**	Page 9
**Table S4**	Page 10
**Table S5**	Page 11
**Table S6**	Page 12

MOLECULAR ECOLOGY

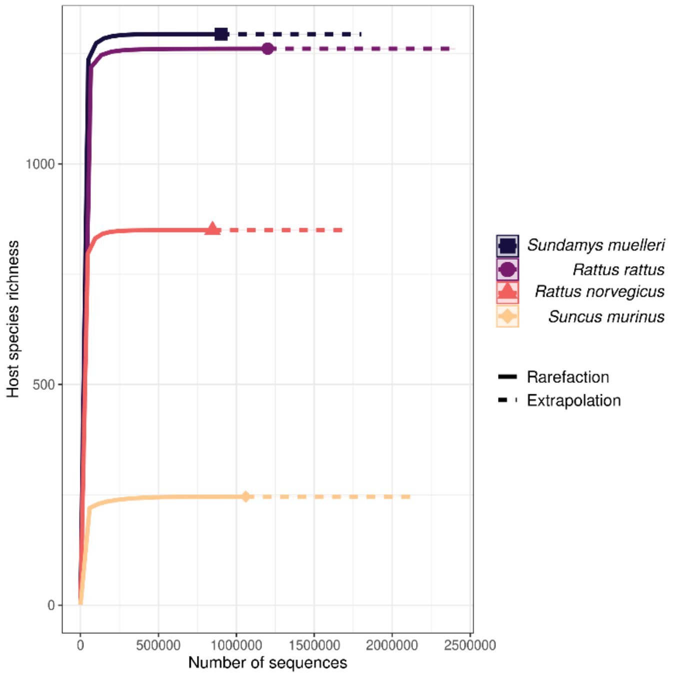

Figure S1: Alpha diversity rarefaction curves based on the cumulative number of amplicon sequence variants (ASV) recorded in fecal samples from the four focal host species. Estimates are based on Hill numbers, solid lines refer to the rarefied samples, dashed lines to extrapolated predictions of the same number of samples.

MOLECULAR ECOLOGY

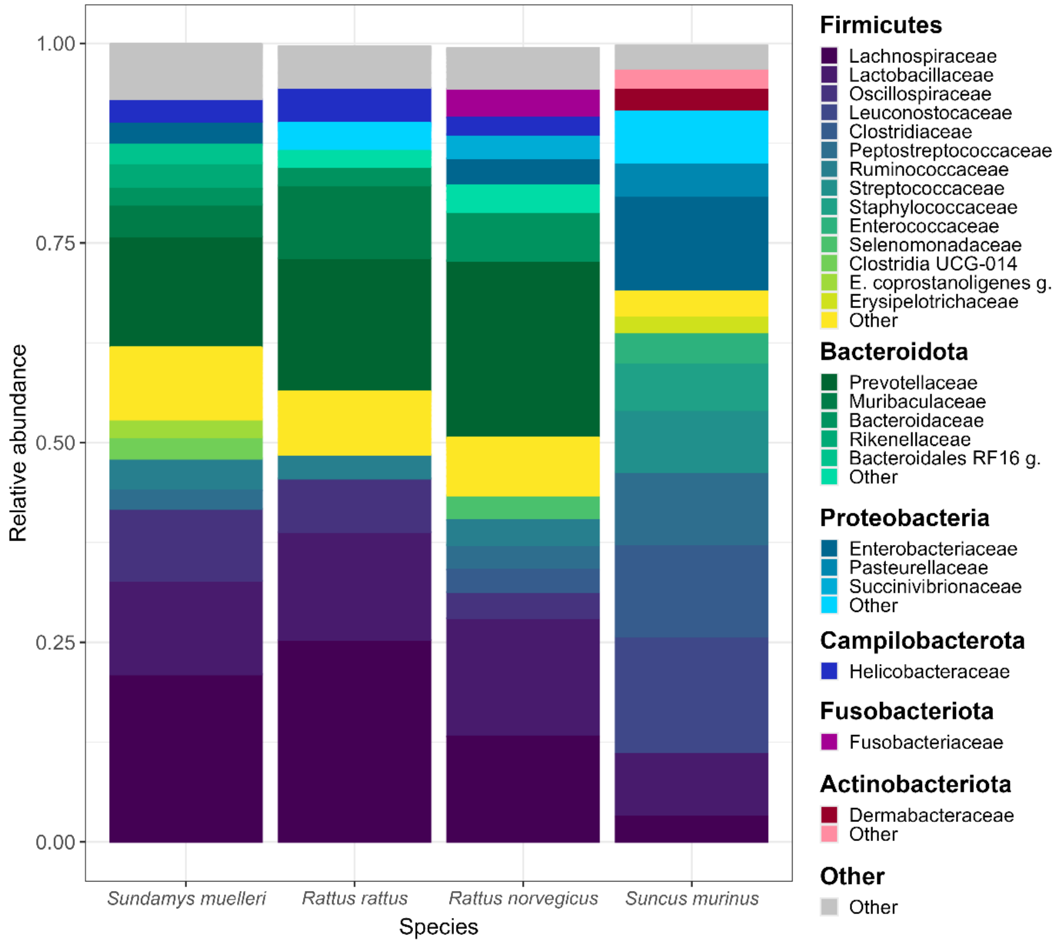

Figure S2: Faecal bacterial composition aggregated at family taxonomic level for four small mammal species from Borneo. Compositions are based on the relative proportion of amplicon sequence variants assigned to different taxonomic groups pooled for all individuals of each host species (Sample sizes: *Sund. muelleri*: n = 58, *R. rattus*: n = 74, *R. norvegicus*: n = 49, *Sunc. murinus*: n = 55). For each host species, families representing less than 2% relative abundance were clustered together as 'Other'.

(E. coprostanoligenes g.: Eubacterium coprostanoligenes group; Bacteroidales RF16 g.: Bacteroidales RF16 group.).

MOLECULAR ECOLOGY

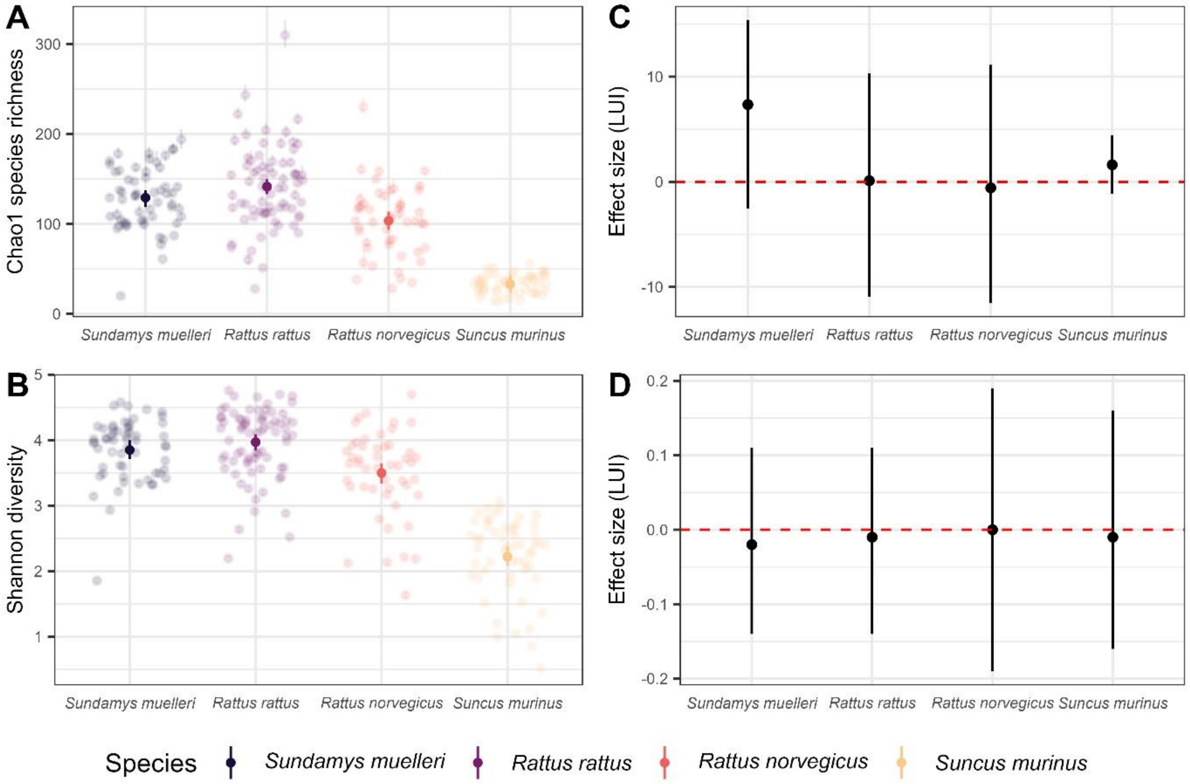

Figure S3: (A and B) Plots of the estimates of relative alpha diversity measures (Chao1 species richness and Shannon diversity) for each host individual, for the four studied small mammal species. Dark point and error bars represent the expected average estimate based on a generalised linear model and an iterative model fit to rarefied data as described in the methods. (C and D) Plots of the coefficient estimates of LUI as linear predictor for the variation in Chao1 and Shannon metrics depicted in A and B. Points represent the mode and lines represent the 95% CI, based on separate generalised linear models and iterative model fits for each species.

MOLECULAR ECOLOGY

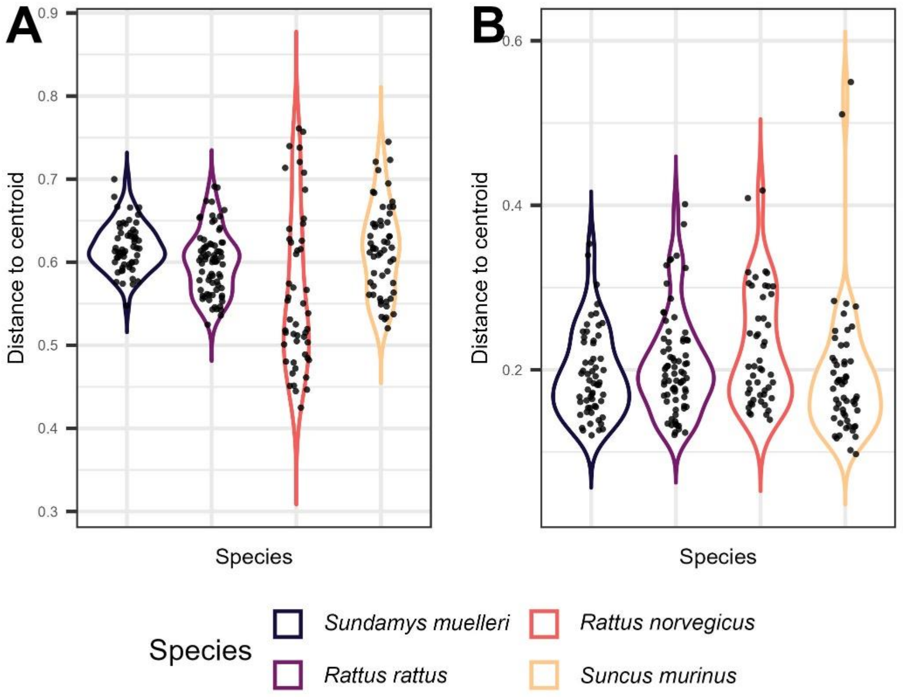

Figure S4: plots of beta dispersion, i.e. distance to species centroid defined using non-metric multidimensional scaling (NMDS), for the four studied small mammal species. (Sample sizes: n = 58 for *Sund. muelleri*, n = 74 for *R. rattus*, n = 49 for *R. norvegicus*, n = 55 for *Sunc. murinus*).

MOLECULAR ECOLOGY

Table S1: Average estimate of alpha diversity (Chao1 species richness and Shannon diversity) and effect of land use intensity (LUI) on bacterial assemblages in host individuals for the four studied small mammal species. Values are reported as mode [95% CI], based on generalised linear models and an iterative model fit to rarefied data as described in the methods.

	**Alpha diversity average estimates**		**LUI effect size**	
**Species**	**Chao1 species richness**	**Shannon diversity**	**Chao1 species richness**	**Shannon diversity**
Sundamys muelleri	129.16 [118.69 – 137.49]	3.85 [3.71 – 4.00]	7.35 [-2.52 – 15.39]	-0.02 [-0.14 – 0.11]
Rattus rattus	141.58 [133.23 – 149.67]	3.97 [3.84 – 4.09]	0.11 [-10.95 – 10.33]	-0.01 [-0.14 – 0.11]
Rattus norvegicus	103.47 [93.30 – 113.70]	3.50 [3.34 – 3.65]	-0.57 [-11.53 – 11.13]	-0.00 [-0.19 – 0.19]
Suncus murinus	33.16 [23.56 – 42.95]	2.22 [2.08 – 2.38]	1.62 [-1.14 – 4.45]	-0.01 [-0.16 – 0.16]

MOLECULAR ECOLOGY

Table S2: Proportion of variance in Bray Curtis and weighted UniFrac beta diversity of microbial assemblages from distinct host species explained by land use intensity (LUI) and spatial proximity of capture locations in Generalised dissimilarity models. Means and 95% confidence intervals of the variance explained are reported for those iteratively fitted models that returned estimates (models did not converge when fitted for both Bray Curtis and weighted UniFrac microbial assemblage dissimilarity in *Rattus rattus*).

**Predictor variable**	**Rat species**		
**Bray Curtis**			
	Sundamys muelleri	***Rattus rattus***	***Rattus norvegicus***
LUI	0.01 [0 – 0.03]	NA	7.14 [6.88 – 7.41]
Spatial proximity	0.38 [0.29 – 0.44]	NA	0
LUI ∩ Spatial proximity	0	NA	0
Unexplained	99.60 [99.50 – 99.70]	NA	92.80 [92.60 – 93.20]
**Weighted UniFrac**			
	Sundamys muelleri	***Rattus rattus***	***Rattus norvegicus***
LUI	0.01 [0.01 – 0.02]	NA	3.50 [3.31 – 3.76]
Spatial proximity	0.16 [0.12 – 0.22]	NA	0

LUI ∩ Spatial proximity	0	NA	0
Unexplained	99.80 [99.80 – 99.90]	NA	96.50 [96.20 – 96.70]

MOLECULAR ECOLOGY

Table S3: Analysis of similarity (ANOSIM) comparing microbiome assemblage of host species based on Bray Curtis and weighted UniFrac dissimilarity measures. The smallest R values and relative host species pairs are reported in bold; the most related species pairs and relative values are underlined.

**Comparisons**	**Bray-Curtis**		**Weighted UniFrac**	
	R value	p-value	R value	p-value
Sundamys muelleri – Rattus rattus	**0.42**	**0.001**	**0.10**	**0.001**
Sund. muelleri – R. norvegicus	0.69	0.001	0.33	0.001
<u>*R. rattus – R. norvegicus*</u>	<u>0.72</u>	<u>0.001</u>	<u>0.29</u>	<u>0.001</u>
Sund. muelleri – Suncus murinus	0.86	0.001	0.81	0.001
R. rattus – Sunc. murinus	0.91	0.001	0.78	0.001
R. norvegicus – Sunc. murinus	0.70	0.001	0.65	0.001

MOLECULAR ECOLOGY

Table S4: results of Tukey's HSD (Honest Significant Difference) test used to compare beta dispersion among host species. The difference in terms of observed means for each pair of species, with 95% CI, and the adjusted p-values are reported on the left for Bray-Curtis and on the right for weighted UniFrac.

	**Bray-Curtis**		**Weighted UniFrac**	
	**Difference in observed means [CI]**	**Adjusted p-value**	**Difference in observed means [CI]**	**Adjusted p-value**
Sundamys muelleri – Rattus rattus	0.02 [0.01, 0.04]	0.25	0.01 [- 0.02, 0.04]	0.75
Sund. muelleri – R. norvegicus	0.05 [0.03, 0.08]	< 0.001 ***	0.03 [0, 0.06]	0.10
R. rattus – R. norvegicus	0.04 [0.01, 0.06]	< 0.01 **	0.02 [- 0.01, 0.05]	0.47
Sund. muelleri – Suncus murinus	< 0.01 [- 0.02, 0.03]	1	< 0.01 [- 0.03, 0.04]	1
R. rattus – Sunc. murinus	0.01 [- 0.01, 0.04]	0.46	0.02 [- 0.02, 0.05]	0.58
R. norvegicus – Sunc. murinus	0.05 [0.02, 0.08]	< 0.001 ***	0.03 [0, 0.07]	0.06

MOLECULAR ECOLOGY

Table S5: Results from generalized linear models indicating the effect of land use intensity on Bray-Curtis dispersion (on the left) and weighted UniFrac dispersion (on the right), for each studied small mammal species.

	**Bray-Curtis dispersion**				**Weighted UniFrac dispersion**			
	**Estimate**	**SE**	**T value**	**p-value**	**Estimate**	**SE**	**T value**	**p-value**
	Sundamys muelleri							
Intercept	0.62	0.01	55.94	< 0.001 ***	0.19	0.02	9.37	< 0.001 ***
LUI	0.01	0.03	0.26	0.80	0.01	0.05	0.29	0.77
	Rattus rattus							
Intercept	0.60	0.02	31.70	< 0.001 ***	0.20	0.03	6.49	< 0.001 ***
LUI	0	0.04	-0.11	0.92	0.02	0.06	0.27	0.79
	Rattus norvegicus							
Intercept	0.74	0.05	13.88	< 0.001 ***	0.29	0.04	6.86	< 0.001 ***
LUI	-0.24	0.07	-3.36	< 0.01 **	-0.10	0.06	-1.64	0.11
	Suncus murinus							
Intercept	0.58	0.03	17.15	< 0.001 ***	0.18	0.05	3.48	< 0.01 **
LUI	0.06	0.06	1.03	0.31	0.03	0.09	0.27	0.79

MOLECULAR ECOLOGY

Table S6: differentially abundant ASVs in relation to LUI identified in the four studied species using ANCOM-BC2 method (*adj p* < 0.05). ASVs that passed the sensitivity test for pseudo-count addition are reported in bold.

(LFC: log fold change; SE: standard error; SS: sensitivity test).

	**Species**	**Host species**	**LFC**	**SE**	**W test**	**Adj p**	**SS**
1	*Prevotellaceae spp.*	*Sundamys muelleri*	-14.39	1.62	-8.88	0.025	FALSE
2	*Desulfovibrionaceae spp.*	*Sundamys muelleri*	-12.40	1.61	-7.68	0.025	FALSE
3	*Clostridium perfringens*	*Sundamys muelleri*	18.35	1.64	11.20	0.050	FALSE
4	*Prevotellaceae UCG-001 spp.*	*Rattus norvegicus*	-8.00	0.74	-10.75	0.005	FALSE
5	*Muribaculaceae spp.*	*Rattus norvegicus*	-7.81	0.72	-10.92	0.005	FALSE
**6**	***Bacteroides sartorii***	***Rattus norvegicus***	**-7.73**	**0.81**	**-9.54**	**< 0.001**	**TRUE**
7	*Holdemanella spp.*	*Rattus norvegicus*	-7.57	0.71	-10.70	0.001	FALSE
8	*Alloprevotella spp.*	*Rattus norvegicus*	-6.66	0.86	-7.73	0.017	FALSE
**9**	***Terrisporobacter spp.***	***Rattus norvegicus***	**-6.65**	**0.71**	**-9.34**	**0.005**	**TRUE**
10	*Bacteroides spp.*	*Rattus norvegicus*	-6.49	0.70	-9.31	0.022	FALSE
11	*Paeniclostridium spp.*	*Rattus norvegicus*	-6.37	0.81	-7.86	0.017	FALSE
12	*Lachnospiraceae spp.*	*Rattus norvegicus*	-5.27	0.75	-7.05	0.013	FALSE
13	*Erysipelotrichaceae spp.*	*Rattus norvegicus*	-5.26	0.75	-7.04	0.036	FALSE
14	*Bacteroides rodentium*	*Rattus norvegicus*	-5.21	0.72	-7.27	0.035	FALSE
15	*Lachnospiraceae NK4A136 group spp.*	*Rattus norvegicus*	-4.91	0.71	-6.88	0.022	FALSE
16	*Eubacterium siraeum group spp.*	*Rattus norvegicus*	-4.88	0.75	-6.52	0.023	FALSE
17	*Muribaculaceae spp.*	*Rattus norvegicus*	-4.71	0.70	-6.72	0.039	FALSE
18	*Treponema succinifaciens*	*Rattus norvegicus*	-4.39	0.94	-4.69	0.010	FALSE
**19**	***Eubacterium coprostanoligenes group spp.***	***Rattus norvegicus***	**-4.15**	**0.75**	**-5.57**	**0.013**	**TRUE**
20	*Prevotellaceae UCG-001 spp.*	*Rattus norvegicus*	-4.14	0.74	-5.61	0.011	FALSE

21	*Blautia spp.*	*Rattus norvegicus*	-4.10	0.73	-5.62	0.022	FALSE
22	*Muribaculaceae spp.*	*Rattus norvegicus*	-4.09	0.69	-5.89	0.011	FALSE
**23**	***Bacteroides spp.***	***Rattus norvegicus***	**-4.07**	**0.97**	**-4.21**	**0.035**	**TRUE**
24	*Rikenellaceae RC9 group spp.*	*Rattus norvegicus*	-4.00	0.85	-4.72	0.013	FALSE
25	*Prevotellaceae UCG-001 spp.*	*Rattus norvegicus*	-3.98	0.83	-4.80	0.008	FALSE
26	*Gastranaerophilales spp.*	*Rattus norvegicus*	-3.88	0.72	-5.42	0.035	FALSE
27	*Lactococcus garvieae*	*Rattus norvegicus*	-3.79	0.76	-4.98	0.041	FALSE
28	*Lachnospiraceae spp.*	*Rattus norvegicus*	-3.56	0.70	-5.11	0.040	FALSE
29	*Prevotellaceae NK3B31 group spp.*	*Rattus norvegicus*	-3.36	0.74	-4.55	0.047	FALSE
30	*Muribaculaceae spp.*	*Rattus norvegicus*	-3.28	0.72	-4.54	0.010	FALSE
31	*CAG-352 spp.*	*Rattus norvegicus*	-3.17	0.73	-4.35	0.019	FALSE
32	*UCG-005 spp.*	*Rattus norvegicus*	-3.08	0.74	-4.14	0.022	FALSE
33	*Lachnospiraceae spp.*	*Rattus norvegicus*	-2.94	0.76	-3.88	0.043	FALSE
34	*Prevotella spp.*	*Rattus norvegicus*	-2.88	0.72	-3.98	0.041	FALSE
35	*Treponema berlinense*	*Rattus norvegicus*	-2.79	0.81	-3.43	0.041	FALSE
36	*Bacteroides vulgatus*	*Rattus norvegicus*	-2.76	0.87	-3.17	0.035	FALSE
37	*Coprococcus spp.*	*Rattus norvegicus*	-2.74	0.71	-3.85	0.043	FALSE
38	*Lachnospira spp.*	*Rattus norvegicus*	-2.73	0.86	-3.18	0.046	FALSE
39	*Muribaculaceae spp.*	*Rattus norvegicus*	-2.68	0.71	-3.78	0.021	FALSE
40	*NK4A214 group spp.*	*Rattus norvegicus*	-2.57	0.71	-3.63	0.043	FALSE
41	*NK4A214 group spp.*	*Rattus norvegicus*	-2.56	0.69	-3.72	0.046	FALSE
42	*Bacteroides spp.*	*Rattus norvegicus*	-2.56	0.70	-3.66	0.043	FALSE
43	*Lachnospiraceae spp.*	*Rattus norvegicus*	-2.49	0.71	-3.52	0.039	FALSE
44	*Oscillibacter spp.*	*Rattus norvegicus*	-2.44	0.70	-3.48	0.035	FALSE
45	*Ruminococcus torques group spp.*	*Rattus norvegicus*	-2.19	0.69	-3.19	0.048	FALSE
46	*Parabacteroides spp.*	*Rattus norvegicus*	-2.08	0.70	-2.99	0.049	FALSE

**47**	***Romboutsia spp.***	***Rattus norvegicus***	**2.09**	**0.71**	**2.96**	**0.046**	**TRUE**
48	*Phascolarctobacterium spp.*	*Rattus norvegicus*	2.22	0.70	3.17	0.046	FALSE
**49**	***Anaerostipes spp.***	***Rattus norvegicus***	**2.42**	**0.71**	**3.42**	**0.048**	**TRUE**
50	*Romboutsia spp.*	*Rattus norvegicus*	2.46	0.88	2.82	0.043	FALSE
51	*Bacteroides spp.*	*Rattus norvegicus*	2.81	0.85	3.29	0.048	FALSE
52	*Bacteroidales RF16 group spp.*	*Rattus norvegicus*	2.82	0.73	3.88	0.023	FALSE
53	*Veillonella spp.*	*Rattus norvegicus*	2.91	0.74	3.92	0.013	FALSE
**54**	***Bacteroides spp.***	***Rattus norvegicus***	**2.97**	**0.71**	**4.20**	**0.029**	**TRUE**
**55**	***Sutterella spp.***	***Rattus norvegicus***	**3.00**	**0.81**	**3.69**	**0.013**	**TRUE**
**56**	***Subdoligranulum spp.***	***Rattus norvegicus***	**3.32**	**0.77**	**4.30**	**0.026**	**TRUE**
**57**	***Collinsella spp.***	***Rattus norvegicus***	**3.71**	**0.84**	**4.41**	**0.022**	**TRUE**
58	*Eubacterium nodatum group spp.*	*Rattus norvegicus*	3.72	0.69	5.37	0.023	FALSE
**59**	***Lactobacillus spp.***	***Rattus norvegicus***	**3.74**	**1.39**	**2.69**	**0.047**	**TRUE**
60	*Lactobacillus spp.*	*Rattus norvegicus*	3.79	0.75	5.04	0.017	FALSE
61	*Fusobacterium sp.*	*Rattus norvegicus*	3.90	0.70	5.56	0.022	FALSE
62	*Succinivibrio spp.*	*Rattus norvegicus*	3.96	0.79	5.01	0.005	FALSE
63	*Prevotellaceae NK3B31 group spp.*	*Rattus norvegicus*	4.10	0.74	5.51	0.035	FALSE
64	*Staphylococcus spp.*	*Rattus norvegicus*	4.17	0.81	5.17	0.005	FALSE
65	*Oscillospiraceae spp.*	*Rattus norvegicus*	4.22	0.70	6.00	0.019	FALSE
66	*Erysipelotrichaceae spp.*	*Rattus norvegicus*	4.48	0.87	5.18	0.017	FALSE
67	*Mogibacterium spp.*	*Rattus norvegicus*	4.81	0.71	6.75	0.022	FALSE
**68**	***Clostridium sensu stricto 1 spp.***	***Rattus norvegicus***	**5.05**	**0.99**	**5.13**	**0.002**	**TRUE**
69	*CAG-352 spp.*	*Rattus norvegicus*	5.12	0.70	7.33	0.035	FALSE
70	*Bacteroides spp.*	*Rattus norvegicus*	5.47	0.71	7.71	0.017	FALSE
71	*Ruminococcus spp.*	*Rattus norvegicus*	5.57	0.71	7.83	0.011	FALSE
**72**	***Eubacterium hallii group spp.***	***Rattus norvegicus***	**6.12**	**0.70**	**8.74**	**0.024**	**TRUE**

73	*Eubacterium coprostanoligenes group spp.*	*Rattus norvegicus*	6.33	0.71	8.94	0.013	FALSE
74	*Helicobacter cholecystus*	*Rattus norvegicus*	6.73	0.69	9.71	0.006	FALSE
75	*Gastranaerophilales spp.*	*Rattus norvegicus*	12.58	0.70	17.90	0.010	FALSE
76	*Alloprevotella spp.*	*Rattus norvegicus*	16.84	0.71	23.70	0.006	FALSE
77	*Weissella spp.*	*Suncus murinus*	-13.97	2.14	-6.54	0.003	FALSE
78	*Turicibacter spp.*	*Suncus murinus*	-8.30	2.03	-4.09	0.011	FALSE
79	*Candidatus Amphibiichlamydia*	*Suncus murinus*	-8.28	2.23	-3.72	0.033	FALSE
80	*Enterobacteriaceae spp.*	*Suncus murinus*	-7.21	2.15	-3.36	0.039	FALSE
81	*Clostridium baratii*	*Suncus murinus*	9.11	2.13	4.28	0.040	FALSE
82	*Clostridium sensu stricto 1 spp.*	*Suncus murinus*	15.26	2.20	6.93	0.033	FALSE
83	*Cellulosilyticum spp.*	*Suncus murinus*	17.64	2.13	8.29	0.003	FALSE
84	*Romboutsia spp.*	*Suncus murinus*	18.68	2.13	8.78	0.003	FALSE
